# Pulmonary embolism after diagnostic curettage in patient with adenomyosis and hysteromyoma: A case report and brief review of literature

**DOI:** 10.1097/MD.0000000000036279

**Published:** 2023-12-01

**Authors:** Zhen Cheng, Min Yan, Yu-Yan Wu, Xin-Ran Li, Xiang-Tao Pan

**Affiliations:** a Hematology Department, Taicang Hospital Affiliated to Soochow University, Taicang City, Jiangsu Province, People’s Republic of China; b Medical College of Soochow University, Soochow City, Jiangsu Province, People’s Republic of China.

**Keywords:** adenomyosis, coagulopathy, curettage, hypercoagulability, hysteromyoma, pulmonary embolism

## Abstract

**Rationale:**

Pulmonary embolism (PE) is a common cause of cardiovascular death whose major acquired risk factors include postoperative states, pregnancy, malignancy, and age. We report a case of PE that occurred after diagnostic curettage for abnormal uterine bleeding, with a medical history of adenomyosis and hysteromyoma.

**Patient concerns and diagnoses:**

A 31-year-old Han Chinese female was referred to our hospital with menstrual disorders, increased menstrual flow, and severe anemia. After admission, the patient was treated with a blood transfusion, iron supplementation, and erythropoietin, and diagnostic curettage was performed the following day. On the first postoperative day, the patient developed pulmonary embolism with dyspnea and fever diagnosed by CT pulmonary angiography and significantly elevated D-dimer.

**Interventions and outcomes:**

Molecular weight heparin was administered for PE for 2 weeks, dyspnea was relieved significantly after 2 days of treatment and the uterine bleeding did not increase; and gonadotropin-releasing hormone agonists were administered for adenomyosis after 1 week of anticoagulant therapy to reduce bleeding. We followed up for 6 months, and the patient had no recurrence of thrombosis and uterine bleeding had improved.

**Conclusion:**

We speculate that the occurrence of pulmonary embolism was closely related to adenomyosis, hysteromyoma, and curettage in this patient. Treating the presence of both menstrual bleeding and thromboembolism is challenging, and careful management is necessary to avoid therapeutic contradictions.

## 1. Introduction

Adenomyosis and hysteromyoma are common benign uterine tumors that occur in 20% to 40% of women during their reproductive years.^[1,2]^ Adenomyosis is caused by endometriosis and often accompanied by periodic bleeding, dysmenorrhea, and infertility.^[3–5]^ As for hysteromyoma, which is caused by excessive secretion of estrogen, mostly grows in the intermuscular space, while those growing under the mucosa may also show increased menstrual flow. Both adenomyosis and hysteromyoma can cause abnormalities of coagulation system.^[6–8]^ Adenomyosis can induce venous thromboembolism in 12.2% of cases.^[9]^ Recently, stroke and pulmonary embolism (PE) associated with these noncancerous gynecologic diseases have been reported.^[9,10]^ However, both rarely develop simultaneously in clinical practice, and PE after curettage is more rare. Here, we report a case of PE occurring in a patient with adenomyosis and hysteromyoma after curettage and present a literature review on the etiology of hypercoagulability.

## 2. Case presentation

A 31-year-old Han Chinese female was admitted to our hospital on December 23, 2019, who had menstrual disorders for 6 years and increased menstrual flow for 2 months who had diagnosed as adenomyosis in other hospital and received no related treatment. The patient had no other underlying diseases and no history of special medications. In the routine physical examination, except for slight sinus tachycardia (123 beats/minutes) and pale appearance due to severe anemia, there were no obvious abnormalities in important organs such as the heart, lungs, and kidneys, and there was no swelling of the limbs. However, a small amount of dull red blood was found in the vagina, and the uterine body was as large as it was at 9 weeks of gestation in the gynecological examination. Doppler ultrasound showed that the size of the uterus was 84 × 88 × 70 mm, the thickness of the endometrium was 8 mm, and a 38 × 32 × 40 mm hypoechoic mass was observed near the bottom of the right posterior wall of the uterus, which was diagnosed as uterine enlargement, adenomyosis, and hysteromyoma. Routine laboratory tests showed that white blood cell and platelet counts were slightly elevated, hemoglobin was significantly lower, serum ferritin was only 10 ng/mL, CA125 was significantly increased (416 U/mL), and other blood parameters such as those associated with liver and kidney function, D-dimer (DD), C-reactive protein (CRP), antinuclear antibodies, and anticardiolipin antibodies were all normal (see Table 1 for details). Uterine bleeding was preferentially considered to be associated with adenomyosis; however, to rule out gynecological malignancies, the gynecologist planned to perform curettage.

**Table 1:** Laboratory results of the patient before and after curettage.

	At the time of admission	In the event of PE	Normal reference range of hospital
WBC (×10^9^/L)	12.9	12.9	3.5–9.5
NE (×10^9^/L)	10.2	11.1	1.8–6.3
LY (×10^9^/L)	1.9	1.1	1.1–3.2
Hb (g/L)	61.0	44.0	120.0–175.0
PLT (×10^9^/L)	392.0	243.0	125.0–350.0
CRP (mg/L)	3.6	200.0	0.0–10.0
PT (sec)	12.1	12.9	9.6–14.3
TT (sec)	17	16.2	14.0–21.0
APTT (sec)	25.1	27.3	23.3–32.5
AT (%)	95.8	74.6	75.0–130.0
FIB (g/L)	2.6	3.8	1.7–4.1
INR	1.03	1.12	0.85–1.25
DD (mg/L)	0.32	29.23	0.0–0.55

APTT = activated partial thromboplastin time, AT = antithrombin, CRP = C-reactive protein, DD = D-dimer, FIB = fibrinogen, Hb = hemoglobin, INR = International Normalized Ratio, LY = lymphocyte, NE = neutrophile granulocyte, PE = pulmonary embolism, PLT = platelet, PT = prothrombin time, TT = thrombin time, WBC = white blood cell.

On admission, the anemia was considered iron-deficient anemia, resulting from chronic blood loss. The patient received blood transfusion, intravenous iron, and subcutaneous injections of 10,000 units of erythropoietin (EPO) per day for 2 consecutive days, and underwent planned surgery at 3:00 pm on December 24. Due to the patient’s fear of surgery, painless curettage was performed after intravenous propofol administration. The operation proceeded smoothly, lasting 10 minutes, and the volume of blood loss was about 10 mL. She experienced continuous uterine bleeding postoperatively. Histopathological examination revealed simple hyperplasia of the endometrium and chronic inflammatory changes in the cervical canal tissue. Therefore, we concluded that uterine bleeding is mainly associated with adenomyosis.

**Figure 1.**
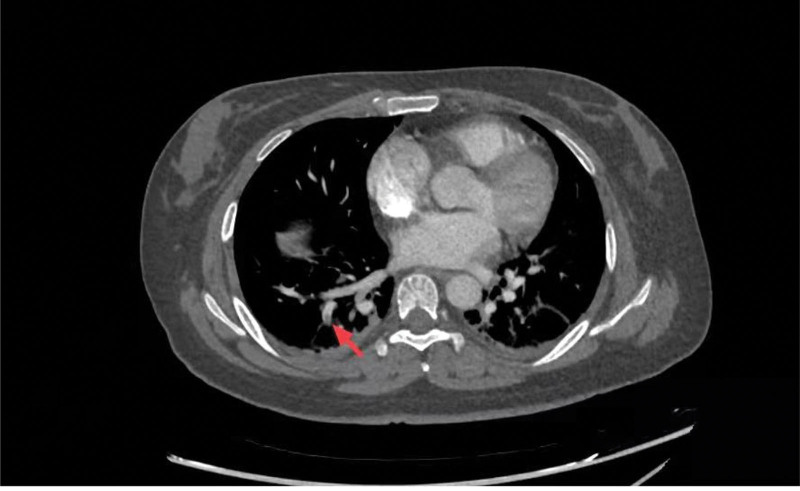
PE shown in the CTPA. The red arrow indicated the site of the emboli was located in right lower pulmonary artery branch embolism. CTPA = CT pulmonary angiography, PE = pulmonary embolism.

At 2:30 pm on December 25, this patient suddenly experienced dyspnea, followed by fever (38.60 degrees Celsius), the oxygen saturation was only 92%, so PE was highly suspected. We conducted CT pulmonary angiography, which showed a right lower pulmonary artery branch embolism (Fig. 1), and both DD and CRP levels significantly increased, severally reached 26.23 g/L and 200.0 mg/L. The diagnosis of acute PE was confirmed. Despite the risk of worsening bleeding, after weighing the pros and cons, we decided to administer anticoagulation with a daily dose of 4000 U of low-molecular-weight heparin for 14 days; however, the bleeding did not increase, and her condition improved rapidly after 2 days. After a week of anticoagulation, DD decreased significantly (1.63 mg/L) and the pulmonary embolism stabilized; therefore, the patient was treated regularly with leuprolide to reduce uterine bleeding. The patient was in good condition in the follow-up of 6 months, there were no occurrence of thrombosis and severe uterine bleeding. The results of laboratory tests on admission and PE are shown in Table 1.

## 3. Discussion

Both adenomyosis and hysteromyoma have major effects on the coagulation system. However, the patient was diagnosed with these 2 gynecologic diseases and presented with excessive menstrual flow. Unexpectedly, this patient developed PE 1 day after curettage, which was confirmed by CT pulmonary angiography and DD. Since the patient had no history of special diseases or medications, her body temperature and CRP were normal when she was admitted, and the patient’s severe anemia could explain her mild tachycardia, suggesting that there was no inflammation before the operation. In addition, although the use of EPO has been reported to cause PE,^[11]^ the patient only received EPO for 2 days before the operation, and the Hb level was still very low; therefore, the possibility of PE caused by EPO is frail. These all show that the patient has no high-risk factors for venous thrombosis before surgery, and when the patient develops PE, CRP is significantly increased (200.0 mg/L) and AT is significantly lower than that at admission (74.6 mg/L vs 95.8 mg/L), so we speculate that the occurrence of PE is closely related to this curettage operation.

Adenomyosis can cause local estrogen excess, progesterone resistance, abnormal uterine contraction, massive neovascularization, and increased expression of inflammatory mediators, leading to hemorrhage or a hypercoagulable state,^[12,13]^ the mechanisms affecting coagulation function are very complicated.^[14]^ Yamanaka et al^[15]^ applied magnetic resonance imaging to adenomyosis patients with thrombotic disease and found that there were microhemorrhage lesions in the ectopic endometrial tissue in the myometrium. Cernogoraz et al^[16]^ performed hysterectomy in a patient with adenomyosis complicated by DIC, and pathology confirmed multiple thrombus lesions in the myometrium. Both of these suggest that there are localized diffuse hemorrhage and fibrin thrombus in the myometrium of patients with adenomyosis We hypothesize that the exposure of endometrial vasculature due to curettage, the entry of fibrin thrombi and related coagulation factors such as tissue factor and fibrinogen into the circulation from microhemorrhagic foci, and surgical emergency (significantly elevation of CRP), contributed to abnormal coagulation function and hypercoagulable state of the body, further causing thrombosis or PE. Our previous studies found significantly increased CRP, decreased AT, and increased FIB levels in many patients with PE,^[17,18]^ which was consistent with the situation in this patient.

Other studies have suggested that factors such as uterine volume, anemia, and CA125 level are also related to coagulation abnormalities in patients with adenomyosis.^[15,19,20]^ Yamanaka^[15]^ suggested that a uterine volume exceeding 100.0 cm^3^ is associated with the risk of activation of the coagulation system, which compresses the iliac vein, resulting in slow blood flow, which puts the body in a hypercoagulable state and leads to thrombosis. This patient had an enlarged uterus, approximately double the size of a normal uterus, which may be associated with the occurrence of PE. Although the patient was severely anemic, the PLT count decreased when PE occurred; therefore, it is unlikely that PE was caused by increased reactive red blood cells and PLT after anemia. Thrombocytosis is thought to be related to iron-deficiency anemia, and the platelet count normalized after treatment with iron. In addition, patients with adenomyosis have been found to have high levels of CA125,^[20]^ which is a mucin molecule that can interact with cells or vascular endothelium and P-selectin on thrombocytes, resulting in platelet-rich microthrombi, thus promoting thrombosis or even PE.^[21]^ In this patient, CA125 level was also significantly elevated, but the patient did not have a gynecologic malignancy confirmed by histologic examination and was considered to be associated with adenomyosis.

Hysteromyomas also have an impact on the coagulation system,^[22,23]^ mainly manifested as increased menstrual flow, while the concurrence of PE is very rare. PE in patients with hysteromyoma reported in the literature mainly occurs after surgery.^[24]^ However, cases of PE after hysteroscopic surgery have also been reported.^[25]^ Most of the possible causes are hysteroscopic surgery prompting the exposure of endometrial vessels and the entry of procoagulant substances such as tissue factor into the blood circulation causing PE, which is similar to the case.

## 4. Conclusion

In conclusion, it is most likely that the PE in this patient was caused by curettage based on the hypercoagulability of adenomyosis combined with hysteromyoma. On the 1 hand, it is due to the abnormal coagulation system and the emergency response of the body caused by the surgery, and on the other hand, the coagulation system was stimulated by the entry of tissue factors into the blood caused by the curettage, and the interaction eventually caused the occurrence of PE. We hope that this case will help enhance the awareness of the link between PE and noncancerous gynecologic diseases and avoid this dilemma.

## Acknowledgments

We are particularly grateful to the gynecologists and anesthesiologists in our hospital who provided us with the help of our article. This work was supported by Suzhou “Science and Education to Promote Health” Youth Science and Technology Program (KJXW2019062); Taicang Science and Technology Program (TC2022JCYL11).

## Author contributions

**Funding acquisition:** Zhen Cheng.

**Investigation:** Min Yan.

**Resources:** Yu-Yan Wu.

**Supervision:** Xiang-Tao Pan.

**Validation:** Xiang-Tao Pan.

**Writing – original draft:** Zhen Cheng, Yu-Yan Wu, Xin-Ran Li.

**Writing – review & editing:** Min Yan.
